# Use of Artificial Intelligence in Undergraduate Medical Student Assessment, Scientific Writing & Peer Review: Promise, Pitfalls, and the Need for Educational Governance

**DOI:** 10.12669/pjms.42.6.19526

**Published:** 2026-06

**Authors:** Kamran Sattar, Mohamad-Hani Temsah, Shaukat Ali Jawaid

**Affiliations:** 1 Kamran Sattar, Assessment & Evaluation Center, College of Medicine, King Saud University, Riyadh, KSA; 2 Mohamad-Hani Temsah, Assessment & Evaluation Center, Pediatric Intensive Care Unit, Department of Pediatrics, College of Medicine, King Saud University, Riyadh, KSA; 3 Shaukat Ali Jawaid, Chief Editor, Pakistan Journal of Medical Sciences, Karachi, Pakistan

## INTRODUCTION

Artificial intelligence (AI), particularly generative AI (GenAI) and large language models (LLMs), is rapidly reshaping the landscape of medical education.^1^ Its influence is no longer limited to information retrieval or content generation. GenAI is now being explored for item writing, MCQ flaw detection, feedback generation, reflective writing assessment, short-answer grading, clinical reasoning support, and academic analytics. For undergraduate medical education, assessment is one of the most sensitive areas for AI integration because assessment decisions directly influence student progression, professional identity formation, patient safety, and public trust.

### Relevance To Pakistan Journal of Medical Sciences Publications and Regional Medical Education:

Recent publications in *Pakistan Journal of Medical Sciences* show that this discussion is both timely and locally relevant. Khan et al. highlighted the potential of ChatGPT to reshape medical education and clinical management, while also emphasizing the need for caution in its use.^2^ More recently, Sabqat et al. explored ChatGPT’s ability to identify and correct MCQ flaws, reporting that it could detect some item-writing problems but struggled with others, including nonparallel options, convergence, and word repetition. The study also found no measurable improvement in overall efficiency after training.^3^ Together, these studies suggest that GenAI may be useful in undergraduate medical assessment, but its role requires careful educational governance.

### The promise of AI in undergraduate medical assessment:

The promise of AI in assessment is understandable. Medical colleges often face challenges related to large student numbers, limited faculty time, variable quality of assessment items, delayed feedback, and the need for continuous quality assurance. AI can assist faculty by reviewing MCQs against item-writing principles, identifying grammatical cues, detecting ambiguous stems, generating formative feedback, supporting blueprinting, and helping teachers refine assessment rubrics. In written assessments, AI may support preliminary scoring, feedback generation, and identification of common misconceptions. In formative assessment, it may help students practice clinical reasoning through structured prompts and receive rapid feedback.

### Efficiency vs Validity:

However, efficiency should not be confused with validity. Assessment in medical education is not simply a process of assigning marks. It is a professional judgement about whether learners are developing the knowledge, skills, attitudes, and ethical values expected of future doctors. Norcini et al. described good assessment in terms of validity or coherence, reproducibility, equivalence, feasibility, educational effect, catalytic effect, and acceptability.^4^ These criteria remain highly relevant when GenAI is introduced into assessment systems. An AI tool may be fast and feasible, but still inappropriate if it does not assess the intended construct, produces inconsistent scores, disadvantages certain groups (due to biased criteria inside the AI algorithm), or weakens meaningful faculty-student interaction.

## KEY CONCERNS IN AI-ASSISTED ASSESSMENT

### Validity and Alignment with Learning Outcomes:

The first major concern is validity. AI-generated or AI-scored assessment must be aligned with curriculum outcomes, clinical competence, and expected levels of undergraduate training. For example, GenAI may produce plausible feedback on a reflective assignment, but the institution must still ask whether it truly evaluates reflection, clinical reasoning, ethical awareness, or merely language quality. Similarly, AI may identify obvious MCQ flaws but miss more subtle threats to validity, such as construct underrepresentation, cueing, inappropriate difficulty, or poor alignment with learning outcomes. Therefore, keeping the human in the loop is vital to ensure GenAI produces valid assessment.

### Reliability, Consistency, and Reproducibility:

The second concern is reliability and consistency. AI systems may generate different responses depending on the prompt, model version, time of use, or system settings. Sabqat et al. reported that ChatGPT’s performance deteriorated during peak hours, which raises practical questions about stability and reproducibility in assessment-related work.^3^ If AI is used in high-stakes assessment, medical colleges and educators must ensure that its outputs are reproducible, auditable, and comparable across student groups and assessment cycles.

### Fairness and Equity:

The third concern is fairness. Undergraduate medical students differ in language proficiency, digital access, socioeconomic background, and familiarity with AI tools. If AI is used for assessment preparation, feedback, or grading, these differences may create new inequities. The *Standards for Educational and Psychological Testing* emphasize validity, reliability, fairness, test administration, and scoring as central issues in educational assessment.^5^ These principles should apply equally to AI-supported assessment. Institutions must ask and monitor whether AI-assisted systems treat students fairly, whether scoring rubrics are transparent, and whether students have equal opportunity to understand how AI is being used.

### Academic Integrity and Assessment Design:

The fourth concern is academic integrity. Generative AI can help students learn, but it can also be used to produce assignments, reflective writing, case summaries, and even answers to open-book or take-home assessments. A purely punitive response is unlikely to succeed. Instead, medical colleges need clear policies that distinguish acceptable, declared, and prohibited uses of AI. Assessment design must also evolve. Greater emphasis may be needed on oral examinations, observed clinical encounters, workplace-based assessment, viva voce linked to written submissions, reflective defense, and assessments requiring local clinical context or personal learning evidence. Some universities have also proposed tiered frameworks for generative AI use in assessment, in which faculty specify the permitted level of AI assistance for each assignment according to intended learning outcomes. Such approaches may improve transparency, provide clearer guidance to students, and support more nuanced governance of AI use across different educational contexts.

### Data Privacy and Accountability:

The fifth concern is data privacy and accountability. Assessment data are sensitive. Student submissions, marks, feedback, examination items, and performance analytics should not be entered into external AI systems without institutional approval, data protection safeguards, and clarity about ownership. The World Health Organization’s guidance on AI for health emphasizes that ethics and human rights must be central to the design, deployment, and use of AI, and that governance should hold stakeholders accountable.^6^ WHO’s later guidance on large multi-modal models also notes that such systems can accept diverse inputs and generate outputs across different formats, which expands both their usefulness and their risk profile in health-related settings.^6^

### From Adoption to Responsible Use:

Therefore, the future of AI in undergraduate medical assessment should not be framed as a choice between adoption and rejection. The more appropriate question is: under what conditions can AI be used responsibly? AI should be considered an assessment assistant, not an autonomous examiner. It may support faculty judgement, but should not replace it, especially in summative or high-stakes decisions.

### A Practical Governance Approach for Medical Colleges:

A practical governance approach may include five levels. First, medical colleges should develop institutional policies defining acceptable AI use in assessment. Second, faculty should receive training in AI literacy, prompt design, limitations of AI-generated outputs, and ethical use. Third, AI-supported assessment tools should be piloted first in low-stakes or formative contexts before any summative use. Fourth, all AI outputs used for assessment should be reviewed by trained faculty. Fifth, institutions should collect local validity evidence, including comparison with human scoring, student perceptions, bias analysis, reliability checks, and impact on learning.

While Artificial Intelligence tools are increasingly being used not only to assist in scientific writing, internal peer review, medical education, and research, but also as assessment tools, it is extremely important that healthcare professionals are properly trained in their intelligent and responsible use, learn prompt engineering while remaining aware of AI limitations. The need for such training is even more important in Low- and Middle-Income Countries like Pakistan. Hence, it is the responsibility of all medical institutions and professional bodies to organize such training sessions for their members.

The Pakistan Association of Medical Editors (PAME), in collaboration with University of Health Sciences, has initiated the Certificate in Medical Editing (CME) and the Certificate in Medical Journalism for Editors (CMJE) programme. It has recently updated and revised its curriculum to include the use of Artificial Intelligence and different AI tools for scientific writing and internal review. PAME also organized a two-day Peer Review Course at University of Lahore in October 2025, where Prof. Mariyah Hidayat shared her personal experience regarding the use of different AI tools for internal review. She pointed out that, in the days to come, “it may not weaken peer review, but it might change the way scientific judgement is formed, making it less independent. In scholarly publishing, it is compensating for a system stretched beyond its own limits.” It has also been observed that reviewers are increasingly relying on AI-generated summaries before undertaking their own interpretation.^7^,^8^

The use of AI tools for scientific writing and peer review, along with their limitations, was again highlighted at the Editors’ Summit held during the 5th International Health Research Conference-2026 at Rehman Medical Institute Peshawar on April 18, 2026. The speakers emphasized that core human values still matter in the AI era**.^9^**

### Implications for Pakistan and Similar Low- and Middle-Income Contexts:

For Pakistan and similar low- and middle-income contexts, the discussion must also include feasibility. Many medical colleges may not have access to secure institutional AI platforms, technical support, or trained assessment units. This does not mean that AI should be ignored. Rather, it means that implementation should be cautious, staged, and context-sensitive. Locally relevant guidelines are needed from medical universities, regulators, and assessment bodies. Collaboration across institutions may help develop shared rubrics, item-review protocols, faculty development modules, and ethical standards for AI use in medical assessment.

### AI as an Assessment Assistant, Not an Autonomous Examiner:

AI has genuine potential to boost undergraduate medical assessment. It can reduce faculty workload, improve feedback timeliness, strengthen item review, and support formative learning. Yet its risks are equally real. Without governance, AI may threaten validity, fairness, professionalism, confidentiality, and academic integrity. The central challenge is not whether AI can assess medical students, but whether medical education systems can govern the evolving AI wisely and continuously enough to protect the purpose of assessment.

In undergraduate medical education, assessment is not merely a technical exercise. It is a social, ethical, and professional responsibility. AI may become a valuable partner in this responsibility, but only when guided by human judgement, educational evidence, and institutional accountability.
